# Frequency of autoimmune thyroiditis in children with Celiac disease and effect of gluten free diet

**DOI:** 10.12669/pjms.36.6.2226

**Published:** 2020

**Authors:** Javaria Rasheed, Rushan Hassan, Muhammad Khalid, Fauzia Zafar

**Affiliations:** 1 Dr. Javaria Rasheed, FCPS (Pediatric Medicine). Senior Registrar, Department of Pediatric Medicine, Unit-1, Nishtar Hospital Multan, Multan, Pakistan; 2 Dr. Rushan Hassan, Post Graduate Trainee, Pediatric Medicine, Department of Pediatric Medicine, Unit-1, Nishtar Hospital Multan, Multan, Pakistan; 3 Dr. Muhammad Khalid, FCPS (Pediatric Medicine), MSc (Epidemiology & Biostatistics). Senior Registrar, Department of Pediatric Medicine, Unit-1, Nishtar Hospital Multan, Multan, Pakistan; 4 Dr. Fauzia Zafar, FCPS (Pediatric Medicine). Professor & Head of Pediatrics Department, Department of Pediatric Medicine, Unit-1, Nishtar Hospital Multan, Multan, Pakistan

**Keywords:** Autoimmune thyroiditis, Celiac disease, Gluten-Free Diet

## Abstract

**Objective:**

To determine the frequency of autoimmune thyroiditis in children with Celiac disease and the effect of gluten free diet on autoimmune thyroiditis.

**Methods:**

We enrolled 100 patients, age 1-12 years of either gender diagnosed as Celiac disease (CD) in this prospective observational study in the Department of Pediatric Medicine, from 1^st^ January 2018 to 30^th^ June 2019. Diagnosis of autoimmune thyroiditis was made if anti–thyroperoxidase >35 iu/ml or anti–thyroglobulin >20 iu/ml at diagnosis of CD and then at one year on gluten free diet (GFD) in all cases. Children with repeat anti-tTG levels > 10 times upper limit normal at 6-months after enrollment were labelled as non-compliant to GFD. Descriptive statistics were used to analyze the data.

**Results:**

Mean age of the participants was 5.94±3.16 years and 53% were females. Fourteen cases of autoimmune thyroiditis were detected at enrollment and six (7%, n/N = 6/86) were later diagnosed on follow-up who were initially negative. Seven hypothyroid cases among the autoimmune thyroiditis were treated with thyroxine and became euthyroid on follow-up testing. Compliance to GFD was 52%. Autoimmune thyroiditis improved on gluten free diet in four cases (28.6%). Of the six euthyroid cases at diagnosis three cases became hypothyroid and all were non-compliant.

**Conclusion:**

Frequency of autoimmune thyroiditis was 20% over a follow-up period of one year. Good compliance with the GFD has some effect on improving autoimmune thyroiditis and maintaining euthyroid status of CD patients.

## INTRODUCTION

Celiac disease (CD) is an immune related disease triggered by the intake of wheat gluten and related prolamines from rye and barley in genetically prone individuals.^1^ Recently, global prevalence of CD is reported to be 1.4% based on serology and 0.7% based on biopsy findings. The prevalence of CD has been reported as 4%, 0.5%, 0.6% and 0.8% in South America, Africa and North America, Asia and Europe and Oceania respectively.^2^

Anti-tissue transglutaminase (tTG) antibody (immunoglobulin IgA) is used as the preferred test to detect CD in children above two years of age. Jejunal or duodenal biopsy must be done when a child has evident clinical signs but negative serological tests after excluding other causes.^3^ The specific therapy for CD is lifelong avoidance of gluten containing food.^4^ CD patients are more likely to develop autoimmune diseases such as autoimmune thyroid disorder, Type-I diabetes mellitus (T1DM), pernicious anemia, autoimmune disease of the liver, sarcoidosis, scleroderma and psoriasis.^5^

Autoimmune thyroiditis (AT) is the commonest thyroid disease of the pediatric age group. Genetic susceptibility contributes to approximately 70% risk of autoimmune thyroiditis in children. It is rare in children below age of one year.^6^ Clinical manifestations of AT vary depending on whether it causes hyperthyroidism or hypothyroidism.^7^ There may be overlap of symptoms of CD and subtle thyroid dysfunction. Therefore, antibodies to thyroglobulin (TG) and thyroperoxidase (TPO) in blood can be detected years before manifestation of autoimmune thyroid disease.^8^

Management of AT associated with CD depends upon thyroid status and mostly it is management of CD with gluten free diet (GFD) rather than AT. Therefore, therapeutic failures may be result of inappropriate management of CD or of thyroid disorder, or both. Early detection by screening and appropriate treatment of autoimmune thyroiditis is the building block of successful CD management.

A recent systematic review identified 34 studies on Celiac disease from Pakistan that included 14 studies in pediatric population and five studies on gluten free diet. None of these studies prospectively assessed the effect of gluten free diet on autoimmune thyroiditis.^9^ The objective of the study was to ascertain the frequency of autoimmune thyroiditis in children with CD and to determine the effect of GFD on autoimmune thyroiditis.

## METHODS

After taking permission from Ethical Review Committee (ERC # 17842-55/NMU), we conducted a prospective observational study at Department of Pediatrics, Nishtar University Hospital Multan from 1^st^ January 2018 to 30^th^ June 2019. A total of 100 children, ages 1-12 years of either gender diagnosed as CD during study period, were included after informed written consent from parents. Enrollment in the study was completed in June 2018 and last follow-up was completed in June 2019. A sample size of 100 patients was calculated by using 15 % prevalence of anti-thyroid antibodies in Celiac disease patients as reported by Ventura A et al.^10^ With 95% confidence level and 7% margin of error.

Diagnosis of Celiac disease was made on the basis of clinical signs and symptoms with laboratory confirmation of serum tTG IgA levels >10 times the upper limit of normal, according to European Society for Paediatric Gastroenterology Hepatology and Nutrition (ESPGHAN) criteria for the diagnosis of CD.^11^ Diagnosis of autoimmune thyroiditis was made on the presence of anti–thyroperoxidase (anti-TPO >35 iu/ml) or anti–thyroglobulin (anti-TG >20 iu/ml) antibodies. Patients with CD who were already on GFD and those with congenital hypothyroidism and with thyrotoxicosis secondary to solitary thyroid nodule and multi-nodular goiter were excluded.

Demographic data including patient’s age and gender was noted. Laboratory tests for thyroid autoimmunity (anti-TPO and anti-TG) at diagnosis and then at one year after putting patient on GFD were done by manual ELISA technique using Eskulisa kit. Compliance to GFD was assessed by tTG (IgA) levels measured after six months and children with tTG – IgA levels > 10 times normal were considered non-compliant. In AT cases, testing with free T_3_ / T_4_ and TSH was done to assess functional thyroid status. The data was entered in and analyzed through SPSS version 23. Age is presented as mean and standard deviation. Median and interquartile range are presented for tTG – IgA levels. Qualitative variables are presented as frequencies and percentages. Chi-square test was used as test of significance for qualitative variables and independent sample t-test for quantitative variables. Mann Whitney U-test was applied for comparison of median.

## RESULTS

During the study period, a total of 100 patients with Celiac disease were enrolled. Mean age of the study participants was 5.94±3.16 years and 53% (n=53) were females. At Celiac disease diagnosis 89% (n=89) of participants were having height < 5^th^ centile and 94% (n=94) had weight < 5^th^ centile. Of all the participants 52% (n=52) were compliant to gluten free diet. Median levels of tTG-IgA antibodies of the participants were 300 IU/ml at initial diagnosis and 160 IU/ml at six months after GFD. A total of 20 cases were diagnosed as autoimmune thyroiditis; 14 at the diagnosis of CD and six cases were detected at one year follow up. The mean age of the children with AT was 8.65±2.4 years compared to 5.2±2.9 years in children without AT (Table-I).

**Table-I:** Demographic characteristics of children with Celiac Disease (N = 100).

Characteristic	Overall (N=100)	Positive anti-thyroid antibodies (n = 20)	Negative anti-thyroid antibodies (n = 80)	p-value
Age (years) (mean ± SD)	5.94 ± 3.16	8.65 ± 2.45	5.26 ± 2.95	<0.001*
***Gender (n, %)***				
Male	47 (47)	9 (45)	38 (47.5)	0.84
Female	53 (53)	11 (55)	42 (52.5)	
***Height centiles (n, %)***				
<5^th^ centile	89 (89)	16 (80)	73 (91.25)	0.15
>5^th^ centile	11 (11)	04 (20)	07 (8.75)	
***Weight centiles (n, %)***				
<5^th^ centile	94 (94)	18 (90)	76 (95)	0.40
>5^th^ centile	06 (06)	02 (10)	04 (05)	
***tTG – IgA levels (median, IQR)***				
On CD diagnosis	300, 100	300, 100	300, 94	0.36^β^
6-month follow up	160, 180	190, 231	160, 173	0.95^β^
***Compliance (n, %)***				
Compliant	52 (52)	09 (45)	43(53.75)	0.48
Non-compliant	48 (48)	11 (55)	37(46.25)	

* Independent sample t-test,

β Mann Whitney U test.

Of 14 patients with AT at CD diagnosis, 8 (57.1%) had anti-TPO positive and 4 (28.5%) were positive for both anti-TPO and anti-TG antibodies. The effects of GFD on thyroid status in 14 children with AT is shown in Table-II. After one year on GFD, five children out of eight with AT remained positive (four compliant with GFD and one non-compliant) and three out of four with AT remain positive for both antibodies. (one compliant who converted from both antibodies +ve to only anti-TPO +ve and 2 non-compliant). At the end of one year, six new cases from previously negative 86 cases were diagnosed as AT in which anti-TPO were positive (4 compliant and 2 non-compliant cases) (Table-II).

**Table-II:** Effect of Gluten Free Diet on thyroid antibodies (n = 20) in children with Celiac Disease (N = 100).

Anti-thyroid antibodies At Diagnosis	1 year after Gluten free diet			

	Previous cases (n = 14)		New Cases (n = 6)	

	Compliant	Non-compliant	Compliant	Non-compliant
Anti-TPO^β^ (n=8)	04	01	04	02
Anti- TG (n=2)	00	02	00	00
Both TPO and Anti-TG (n=4)	01 – only TPO +ve	02	00	00

*Anti-TPO - Thyroid-peroxidase antibodies, Anti TG – Anti-thyroglobulin antibodies

β Three cases became negative after 1 year of GFD.

Regarding the thyroid functional status; out of initial 14 with AT, six were euthyroid, seven hypothyroid and one was hyperthyroid. All seven hypothyroid and one hyperthyroid patient (on treatment) became euthyroid at the end of one-year follow-up. At the end of one year, three out of six euthyroid cases became hypothyroid and these were non-compliant. Out of six newly diagnosed AT cases, two were euthyroid and compliant with GFD, and of the four non-compliant cases, three were euthyroid and one was hypothyroid (Table-III).

**Table-III:** Effect of Gluten Free Diet on thyroid function tests (n = 20) in children with Celiac Disease (N = 100).

Thyroid status on enrollment	*Thyroid status 1 year after Gluten free diet			

	Compliant		Non-compliant	

	Euthyroid	Hypothyroid	Euthyroid	Hypothyroid
Euthyroid (n=6)	03 (50)	-	-	03 (50)
Hypothyroid (n=7)	All patients received thyroxine and became Euthyroid			
Hyperthyroid (n=1)	01 (100)	-	-	-
New cases (n=6)	02 (33.3)	-	03 (50)	01 (16.7)

* n, (%)

## DISCUSSION

Our study determined 20 % frequency of autoimmune thyroiditis and documented the 28.6% improvement in autoimmune thyroiditis over a period of one year follow up on gluten free diet (GFD). Of the twenty AT cases 14 were detected at the diagnosis of CD and 6 on GFD at one year follow up. In a study by Aziz DA et al on 66 children with classic and non-classic CD found that only 2 (3.03%) had autoimmune thyroiditis.^12^ However, the studies conducted by Meloni A et al and Kalyoncu D et al found higher percentage of AT (10.5%) among 324 patients with CD and 16.4% (11/67) of children with CD, respectively.^13^,^14^ The high risk of CD patients to develop AT is explained by the fact that the Celiac disorder carries one or more MHC or other immune related genes supporting AT.

In our study, children with AT were older compared to those without AT (p-value < 0.001), with no major difference in gender frequency. It may be assumed that the older children who had longer duration of exposure to gluten developed AT compared to those who were younger with lesser duration of exposure. Diamanti A et al. reported that duration of GFD differed notably in Celiac patients with AT in contrast to those without AT (7.9±0.9 and 10.2±0.3 years) but no considerable variation was observed for weight and height achievement (1.8±1 vs 3.7±1.5 and 2±1 kg/year vs 4±1 cm/year, respectively).^15^

In our study, eight out of 14 cases with AT (57.1%) were anti-TPO +ve at the start of study. At the end of one year, six new cases appeared despite being on GFD and out of those six new cases, four were anti-TPO +ve. This shows that compliance with GFD had no impact on the presence of any particular type of antibody. In a study, Butt T et al. reported in that anti-TPO antibodies were positive in 16% patients of CD.^16^ In another study by Hakanen M et al., the frequency of anti-TPO and anti-TG was 11.4% and 8.8% respectively.^17^ In the present study, presence of anti TPO antibodies had mixed effect on thyroid functional status. Out of initial 14 patients with AT, six were euthyroid, seven hypothyroid and 1 was hyperthyroid. This could be explained by the fact that anti TPO antibody is an early marker of autoimmunity so it appears before the development of functional thyroid dysfunction. The study by Meloni A et al. reported that out of 34 patients ,28 patients were euthyroid and 6 were hypothyroid.^13^ In Italy, Cassio A et al. reported that out of 31 CD patients with AT, 23 (74%) remained euthyroid during the follow up and eight (26%) had subclinical hypothyroidism.^18^

Though a number of previous studies have shown that duration of gluten intake in Celiac disorder does not relate with the possibility of developing autoimmune illness and that gluten avoidance does not give protection from other auto-immune illnesses,^8^,^18^ numerous reports proposed that strict compliance with GFD was linked with decreased chances of developing subsequent autoimmune disorders and anti-thyroid antibodies.^19^,^20^ Guariso et al. have concluded that GFD produces favorable results on the pre-existing clinical auto-immune disorder and may prevent the development of new autoimmune illness.^21^

### Limitations of the study

Level of thyroid related autoantibodies may reflect the spontaneous fluctuations and transitory normalization in the disease course thus requiring longer duration of follow-up with higher number of patients.

## CONCLUSION

There is increased occurrence of autoimmune thyroiditis in children with Celiac disease. Although good compliance with the GFD has no obvious relationship with the presence or absence of thyroid autoimmunity yet it has clear influence on maintaining euthyroid status of CD patients. Larger, prospective studies with long follow-up are required to explain the clinical importance of anti-thyroid antibodies in children with CD and the influence of GFD on developing autoimmune thyroid disorder.

### Author`s Contribution:

**JR:** Conception of idea, Manuscript writing, Data collection. Also, responsible and accountable for the accuracy / integrity of the work

**RH:** Data collection, Literature review.

**MK:** Data analysis, Statistical analysis, Critical analysis of manuscript.

**FZ:** Final approval and guarantor of the study.
